# The Onconeural Antigen cdr2 Is a Novel APC/C Target that Acts in Mitosis to Regulate C-Myc Target Genes in Mammalian Tumor Cells

**DOI:** 10.1371/journal.pone.0010045

**Published:** 2010-04-07

**Authors:** Kevin J. O'Donovan, Jennifer Diedler, Graeme C. Couture, John J. Fak, Robert B. Darnell

**Affiliations:** Laboratory of Molecular Neuro-Oncology, Howard Hughes Medical Institute and The Rockefeller University, New York, New York, United States of America; UIC, United States of America

## Abstract

Cdr2 is a tumor antigen expressed in a high percentage of breast and ovarian tumors and is the target of a naturally occurring tumor immune response in patients with paraneoplastic cerebellar degeneration, but little is known of its regulation or function in cancer cells. Here we find that cdr2 is cell cycle regulated in tumor cells with protein levels peaking in mitosis. As cells exit mitosis, cdr2 is ubiquitinated by the anaphase promoting complex/cyclosome (APC/C) and rapidly degraded by the proteasome. Previously we showed that cdr2 binds to the oncogene c-myc, and here we extend this observation to show that cdr2 and c-myc interact to synergistically regulate c-myc-dependent transcription during passage through mitosis. Loss of cdr2 leads to functional consequences for dividing cells, as they show aberrant mitotic spindle formation and impaired proliferation. Conversely, cdr2 overexpression is able to drive cell proliferation in tumors. Together, these data indicate that the onconeural antigen cdr2 acts during mitosis in cycling cells, at least in part through interactions with c-myc, to regulate a cascade of actions that may present new targeting opportunities in gynecologic cancer.

## Introduction

Cerebellar-degeneration-related antigen-2 (cdr2) is a target antigen in paraneoplastic cerebellar degeneration (PCD), one of several immune-mediated paraneoplastic neurologic degenerations (PND) that develop as a remote effect of systemic cancers [1], [2]. In the PNDs, onconeural antigens, which are normally expressed in immune-privileged neurons, become ectopically expressed in tumors. PND patients typically present with neurological symptoms while their associated tumors are usually detected subsequently, a phenomenon believed to relate to tumor immune-suppression [3]. It is believed that after the onset of this appropriate tumor immune response, the immune system becomes competent to target onconeural antigen-expressing neurons.

PCD patients harbor breast or ovarian tumors [4] that ectopically express cdr2, which is normally made in cerebellar Purkinje neurons and brainstem neurons and testes [3], [5]. High titer antibodies reactive with cdr2 are found in the serum and cerebrospinal fluid (CSF) of PCD patients and were used to clone several candidate genes [6]–[8]. The only one of these genes expressed at the protein and RNA level in tumors obtained from PCD patients as well as in Purkinje neurons is cdr2 [5]. It is not clear why tumor cells express onconeural antigens, since it appears to put them at risk for immune-mediated destruction. For example, patients with PCD harbor cdr2-specific CD8+ T cells [9], [10]. We previously reported that the PCD antigen cdr2 is commonly expressed in gynecologic cancers in more than 50% of ovarian tumors and 22% of breast tumors obtained from the general population of cancer patients [4]. In addition, we have found that cdr2 interacts with c-myc in the cytoplasm of Purkinje neurons and that cdr2 can inhibit c-myc-dependent transcription in tumor cell lines [11]. These observations suggested a possible role for cdr2 in cancer cell biology.

To explore these observations further, we analyzed cdr2 expression in tumor cells and discovered that it is cell cycle regulated, with protein levels peaking during mitosis. Cdr2 is degraded by the proteasome during mitotic exit by a mechanism that includes recognition and ubiquitination by the anaphase-promoting complex/cyclosome (APC/C). We extend previous observations demonstrating that cdr2 co-localizes and co-precipitates with c-myc in the brain to show that it also does so during mitosis, and that cdr2-mediated modulation of c-myc-dependent transcription is maximal as cells passage through mitosis. Further, we show that cdr2 is required for proper execution of mitosis, as cdr2 knockdown cells have an increased incidence of aberrant mitotic spindles. Cdr2 knockdown cells also exhibit impaired proliferation, while cdr2 overexpression drives proliferation in tumors. Taken together, these data demonstrate a role for cdr2 in mitosis in cycling cells, and suggest that this onconeural antigen may play a functional role in gynecologic tumors.

## Results

### Cdr2 is expressed during mitosis

**Figure 1:**
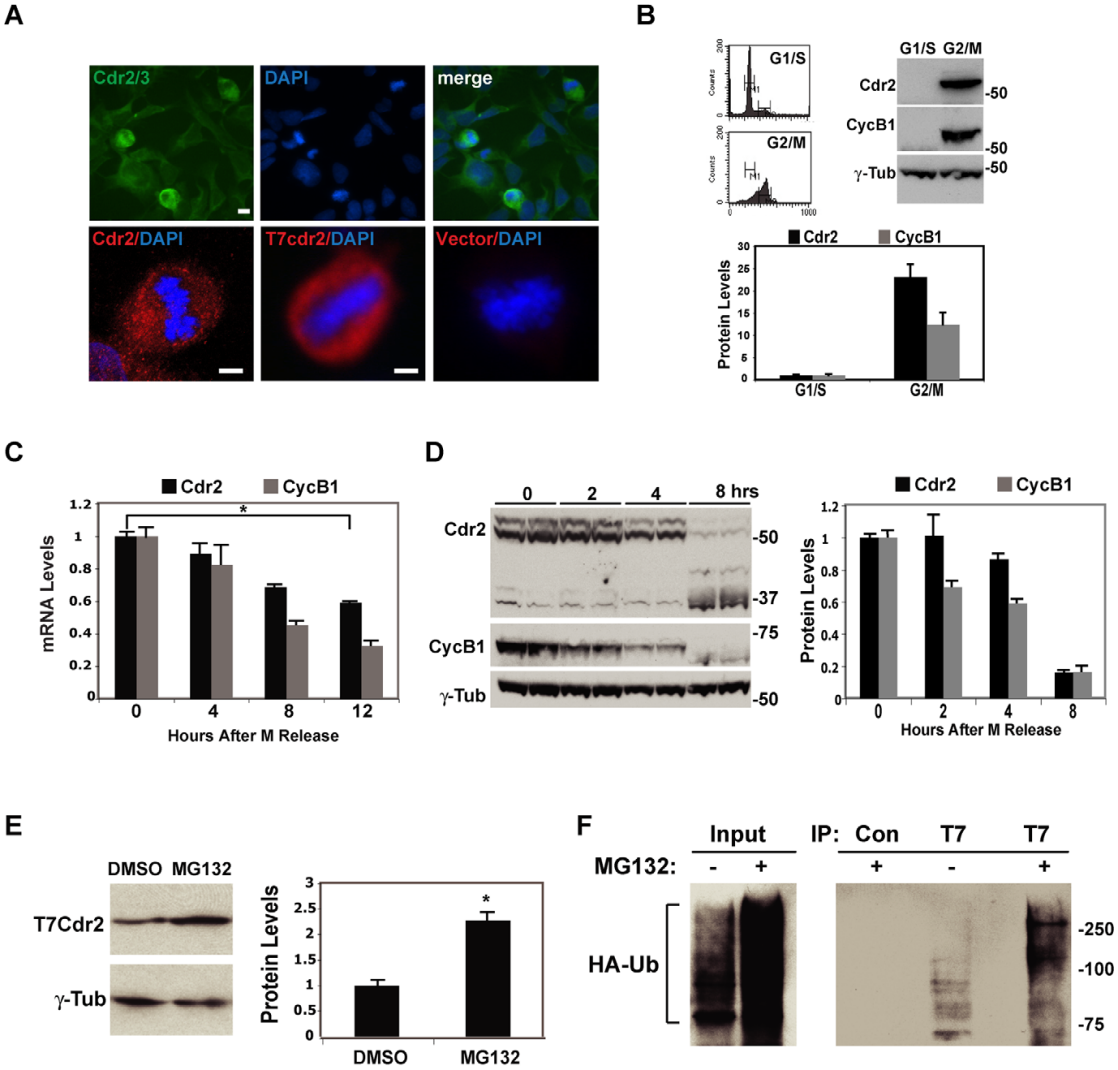
Cdr2 expressed during mitosis, ubiquitinated and degraded during mitotic exit. A. Upper panels, immunostain of HEK293s with cdr2/3 PCD patient CSF (green). Lower left, confocal image of HeLa cells stained with anti-cdr2 (4F5). Lower middle, right, NIH3T3 cells stained with anti-T7 either stably infected with T7cdr2 (lower middle) or vector (lower right); DAPI, all panels except upper left. Scale bars = 20 µm. B. Upper left, flow cytometry of G1/S- and G2/M-arrested HEK293s. Upper right, western blots of HEK293 G1/S and G2/M extracts probed with anti-cdr2 (NB110; top), cyclinB1 (middle) and γ-tubulin (bottom). Bottom panel, cdr2 and cyclinB1 protein quantitation, normalized here and in D to γ-tubulin levels; *p<0.001. C. cdr2 (black) and cyclinB1 (gray) mRNA levels in HeLa cells at 0, 4, 8 and 12 hours after mitotic release, measured by qRT-PCR (normalized to β-actin mRNA). D. Left, western of HEK293s at 0, 2, 4 and 8 hours mitotic release probed with cdr2 (4F5; top), cyclinB1 (middle) and γ-tubulin (bottom) antisera. Right, quantitation of cdr2, cyclinB1 levels. E. Left, western of HEK293 extracts transfected with T7cdr2, treated with DMSO or MG132 probed with T7 and γ-tubulin antisera; right, quantitation of T7cdr2 levels normalized to γ-tubulin. *p<0.005. F. Western blots of input (left) and anti-T7 or control (GFP) immunoprecipitates (right) from HEK293s transfected with T7cdr2 and HA-ubiquitin (HA-Ub) treated with DMSO or MG132, and probed with anti-HA.

We analyzed cdr2 expression in HEK293 cells by immunofluorescence microscopy using PCD patient CSF that specifically recognizes cdr2 and a closely related family member, cdr3 [4], [5], [7], [12]. Only a subset of HEK293 (Fig. 1A) cells exhibits high cdr2/3 expression levels. Counterstaining with the nuclear stain DAPI revealed that these cells are in mitosis. Confocal microscopy with a cdr2-specific monoclonal antibody confirmed that cdr2 is expressed in mitotic cells with a diffuse distribution not contiguous with DNA (Fig. 1A). We found a similar pattern of staining in cells transduced with retroviral constructs expressing T7-tagged cdr2 (Fig. 1A) but not in cells expressing vector alone. Cdr2 protein expression levels appear highest in cells that display a rounded-up appearance typical of mitotic cells (Fig. 1A), while neighboring interphase cells exhibit low-level immunoreactivity.

To directly measure cdr2 protein levels during the cell cycle, we monitored protein levels by Western blot analysis of synchronized cells. We blocked HEK293 cells at G1/S with sequential thymidine and aphidicolin treatment [13], [14], or at metaphase by releasing G1/S-arrested cells into demecolcine (Fig. 1B). Cdr2 immunoreactivity was ∼23-fold higher during G2/M than at G1/S, compared to a ∼12-fold increase in cyclinB1, a protein known to exhibit high expression in mitosis [15]. There was no significant change in the levels of γ-tubulin, a protein whose levels are not cell cycle regulated (Fig. 1B).

To determine whether changes in cdr2 mRNA levels might contribute to the observed changes in cdr2 protein expression, we harvested RNA from HeLa cells at different time points during the cell cycle and measured cdr2 mRNA levels by quantitative RT-PCR (qRT-PCR). As controls, we measured cyclinB1 mRNA levels, as these are known to decrease after mitotic exit [15], and β-actin mRNA levels, as these do not change during the cell cycle [16]. After release from a G2/M block, both cyclinB1 mRNA and cdr2 mRNA levels decreased, with cdr2 levels declining 40% in 12 hours (Fig. 1C). We also observed a significant increase in cdr2 mRNA levels in cells released from G1/S blockade prior to entry into mitosis (Fig. S1). The regulation of cdr2 mRNA levels is consistent with the results of two microarray studies, in human primary fibroblasts [17] and HeLa cells [16] that screened for cell cycle regulated transcripts. In particular, the HeLa study found that cdr2 mRNA levels peaked in G2 and were 3.4-fold higher during early G2 relative to G1, compared with a 7.3-fold increase in cyclinB1 mRNA. Thus, regulation of steady-state cdr2 mRNA during the cell cycle parallels the rise and fall of cdr2 protein during mitosis, suggesting that de novo translation of cdr2 mRNA contributes to the increase in protein.

### cdr2 protein is targeted for degradation upon mitotic exit

To examine whether the high levels of cdr2 in mitosis are subsequently reduced by protein turnover, we assayed whether cdr2 protein was targeted for degradation as cells exit mitosis. HEK293 cells were released from a mitotic block [18], [19] and harvested for Western blot analysis following exit from mitosis. Cdr2 protein levels remain unchanged at 2 hours, but then fell rapidly, such that they were significantly decreased (by ∼80%) 8 hours after mitotic release (Fig. 1D). CyclinB1 levels also decreased sharply following release from the block [15], while γ-tubulin levels remained unchanged under all conditions (Fig. 1D). Notably, at the 8-hour time point, faster-migrating cdr2 immunoreactive bands appeared (Fig. 1D). We interpreted these bands to be C-terminal cdr2 degradation products as they are detectable in proportion to the decrease in full-length cdr2 levels and were specific to C-terminal cdr2 antibodies. Consistent with the rapid degradation observed during mitotic exit, cdr2 has a short half-life of ∼1 hour in HeLa cells (Fig. S1). These studies were performed using cycloheximide treatments since the lack of a specific cdr2-immunoprecipitating antibody precluded us from performing traditional pulse-chase assays. Taken together, these data indicate that cdr2 is regulated during the cell cycle such that protein expression is high throughout mitosis and is specifically degraded as cells exit mitosis.

### cdr2 ubiquitination and degradation

To determine whether cdr2 protein is degraded by the proteasome, we examined the effect of MG132, a proteasome inhibitor, on levels of T7cdr2 in HEK293 cells. MG132 treatment for 6 hours led to a 2-fold increase in T7cdr2 protein levels compared to vehicle-treated cells (Fig. 1E). To determine whether cdr2 degradation may be ubiquitin-mediated, we transiently co-transfected HEK293 cells with T7cdr2 and HA-ubiquitin expression vectors [20]. We treated asynchronous cells with either vehicle or MG132 followed by immunoprecipitation with anti-T7 or control antibodies (Fig. S1), and assayed immunoprecipitates for HA-immunoreactivity. Anti-HA western blotting (Fig. 1F) of control- or MG132-treated inputs confirmed the efficacy of the MG132 in this experiment. In vehicle-treated cells, we observed T7cdr2-ubiquitin conjugates ranging from 75–100 kDa (Fig. 1F), while no HA-ubiquitin was detected in control immunoprecipitations. In the presence of MG132, we observed an increase in total immunoprecipitated T7cdr2-ubiqiutin conjugates (data now shown) as well as an increase in the size of the T7cdr2-ubiquitin species which ranged from ∼150–275 kDa (Fig. 1F).

### cdr2 KEN and destruction boxes are necessary for efficient degradation

**Figure 2:**
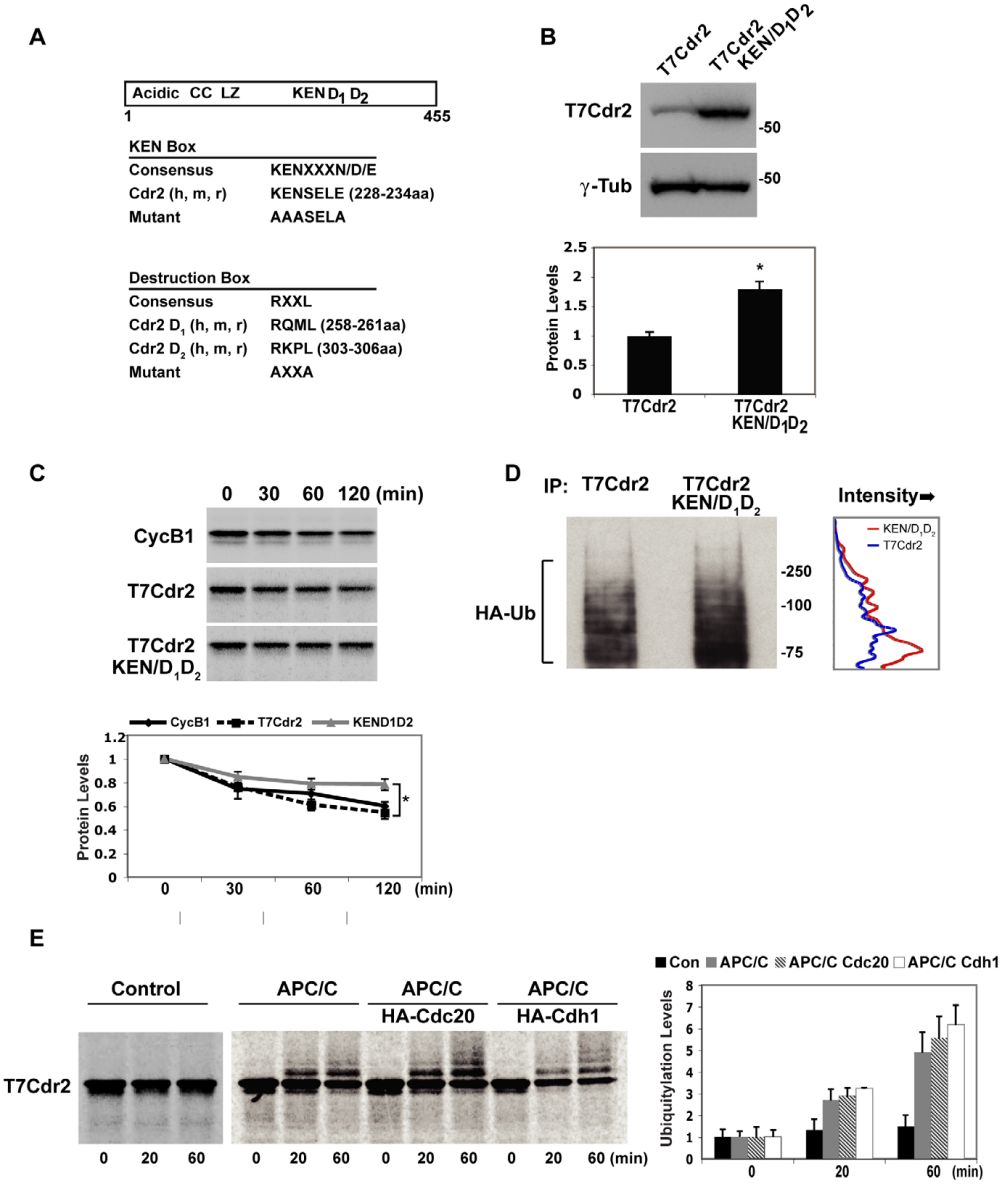
KEN and D box mutants stabilize Cdr2. A. Cdr2 schematic depicting acidic, coiled coil (CC) and leucine zipper (LZ) motifs and KEN and D (D1, D2) boxes. Lower panels, alignment of consensus KEN and D box motifs with human (h), mouse (m) and rat (r) cdr2 and mutants. Amino acid residue numbers are from human Cdr2. B. Upper, HEK293s transfected with T7cdr2 or T7cdr2 KEN/D1D2, released from G2/M block and blotted with T7 or γ-tubulin antisera. Lower, quantitation of T7cdr2 and T7cdr2 KEN/D1D2 levels normalized to γ-tubulin. *p<0.01. C. Upper, autoradiographs of degradation assays of ^35^S-labeled cyclinB1, T7cdr2 and T7cdr2 KEN/D1D2 at indicated times. Lower, quantitation of raw cyclinB1, T7cdr2 and T7cdr2 KEN/D1D2 levels. *p<0.05. D. Left, anti-HA blot of T7 immunoprecipitates from HEK293s transfected with T7cdr2 or T7cdr2 KEN/D1D2 and HA-Ub (see Fig. S2); right, line graph of band intensity (x-axis) for adjacent western blot and molecular weight (y-axis); T7cdr2 (blue), KEN/D1D2 (red). E. Left, autoradiographs of ^35^S-T7cdr2 ubiquitination reaction with the APC/C, APC/C/HACdc20, APC/C/HACdh1, or control (Protein A beads) for indicated times. Right, quantitation of T7cdr2-Ub conjugates.

Ubiquitin-mediated degradation of mitotic B-type cyclins by the anaphase-promoting complex/cyclosome (APC/C) was first described in budding yeast [21], [22]. The APC/C recognizes target proteins harboring sequence elements termed destruction (D) and KEN box motifs [23]. Examination of cdr2 revealed the presence of amino acids matching consensus KEN (KENXXXN/D/E) and D (RXXL) box motifs (Fig. 2A) clustered within an 80 amino acid region in the C-terminal half of the protein. The 80 amino acid region containing cdr2's putative KEN and D boxes is highly conserved, with 89% identity among all available mammalian genomes, and 99% identity in mouse, rat, and human. To test whether mutating these sequences altered cdr2 stability in cells exiting mitosis, we compared the steady-state levels of wild type and a mutant form of T7cdr2 (T7cdr2 KEN/D1D2) in which the consensus KEN and D box residues were mutated to alanines (Fig. 2A) as such mutations have been shown to abrogate KEN and D box recognition [23], [24]. We observed that T7cdr2 KEN/D1D2 protein is expressed at 1.8-fold higher levels than wild type T7cdr2 in HEK293 cells four hours following release from a nocodazole block (Fig. 2B). In parallel, we performed degradation assays in which we incubated *in vitro* synthesized ^35^S-labeled proteins with an HEK293 extract harvested from cells exiting mitosis. After two hours of incubation, T7cdr2 protein was significantly more degraded than T7cdr2 KEN/D1D2 (Fig. 2C; 40% degradation for wild type compared to 20% degradation for KEN/D1D2; (p<0.05), while cyclinB1 was degraded at the same rate as T7cdr2.

We also compared ubiquitination of wild type and T7cdr2 KEN/D1D2 in cells exiting mitosis. We observed robust polyubiquitination of wild type T7cdr2 in the absence of any proteasome inhibition (Fig. 2D). In contrast to wild type T7cdr2, lower molecular weight (∼75KDa) ubiquitin conjugates were evident in T7cdr2 KEN/D1D2 mutant extracts, which may correspond to mono- or di-ubiquitinated cdr2 (quantitated by densitometry; Fig. 2D). An apparent increase in the overall levels of T7cdr2 KEN/D1D2 ubiquitination was parallel to the increase in steady state levels of the T7cdr2 KEN/D1D2 protein (Fig. 2B and data not shown). Taken together, these data are consistent with enhanced stability of T7cdr2 KEN/D1D2 in cells exiting mitosis, and suggest that the cdr2 KEN and D boxes are required for efficient APC/C-mediated polyubiquitination and proteasomal cdr2 degradation in cells exiting mitosis.

### The APC/C ubiquitinates cdr2 *in vitro*

To directly test whether cdr2 is a target of the APC/C, we immunoprecipitated the APC/C from synchronized HEK293 cells exiting mitosis and incubated the complex with *in vitro* translated ^35^S-T7cdr2. We confirmed the efficacy of the APC/C immunoprecipitation by blotting for three APC/C components: cdc27, cdc23 and APC11 (Fig. S2). In the presence of the APC/C, we observed robust ubiquitination of T7cdr2 within 20 minutes (∼3 fold over baseline levels), which increased further by 60 minutes (∼5 fold), with a reciprocal decrease in full length T7cdr2 as the reaction progressed (Fig. 2E). T7cdr2 was not ubiquitinated in the absence of added APC/C. We further demonstrated the specificity of this assay, showing that the APC/C ubiquitinated the canonical substrate cyclinB1, but not an unrelated onconeural antigen, Nova1 (Fig. S2).

From metaphase until the end of G1, cdc20 and cdh1 sequentially activate the APC/C promoting binding to and ubiquitination of D and KEN box-containing proteins, targeting them for proteasomal degradation (/reviewed in/ [25]). To test whether the APC/C co-activators cdc20 and cdh1 could enhance APC/C-mediated ubiquitination of T7cdr2, immunoprecipitated HACdc20 or HACdh1 (Fig. S2) were added to the *in vitro* APC/C ubiquitination reaction. We found that addition of either cdc20 or cdh1 enhanced APC-mediated ubiquitination of T7cdr2 by 10–20% (Fig. 2E). Consistent with our interpretation that the T7cdr2 KEN/D1D2 mutant is ubiquitinated less efficiently in cells (Fig. 2D), we observed that the while the APC/C could still ubiquitinate the KEN/D1D2 mutant *in vitro*, it did so at reduced levels (data now shown). Taken together, these data indicate that cdr2 levels accumulate upon entry into mitosis and levels are then tightly controlled by turnover during mitotic exit by APC/C-induced polyubiquitination and proteasomal degradation.

### Cdr2 is important for proper spindle formation

**Figure 3:**
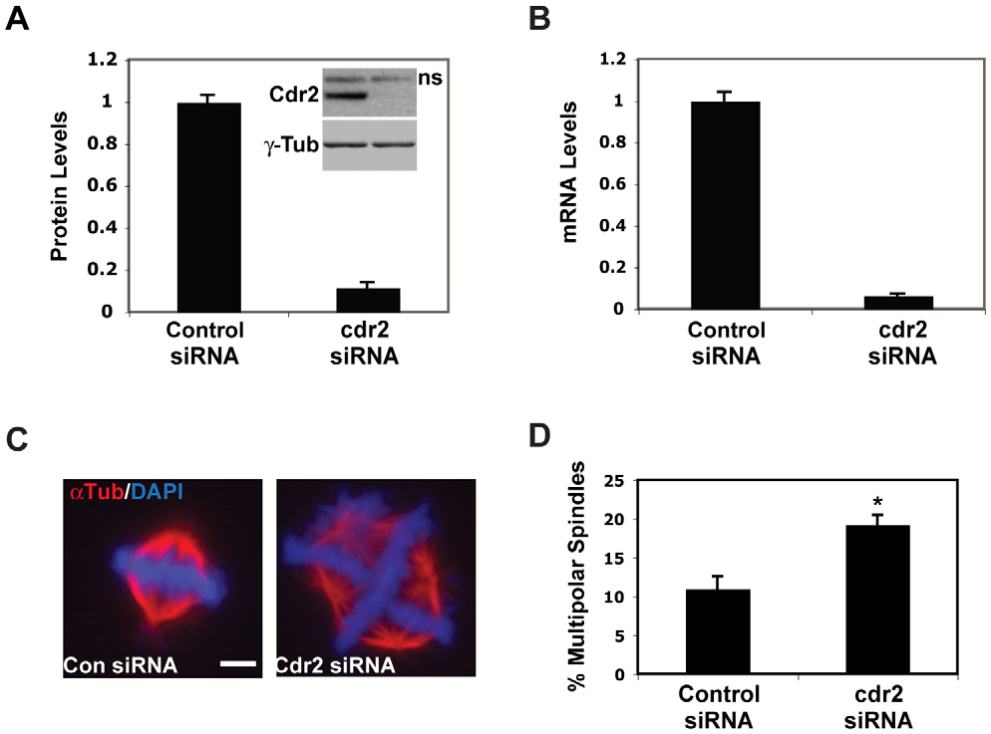
Multipolar spindle defects in cdr2 knockdown cells. A. Quantitation of cdr2 protein normalized to γ-tubulin in HeLa extracts transfected with control or cdr2 siRNAs probed with anti-cdr2 (4F5) and anti-γ-tubulin (inset). B. qRT-PCR of cdr2 mRNA normalized to β-actin mRNA in control or cdr2 siRNA treated cells. C. Immunostain of mitotic control (left) and cdr2 knockdown (right) HeLa cells with α-tubulin antisera and DAPI; Scale bar = 20 µm. D. Quantitation of multipolar spindles in control and cdr2 knockdown cells.

Based on the mitotic-enriched expression and cell cycle regulation of cdr2 we hypothesized the cdr2-deficient HeLa cells may be impaired in the proper execution of mitosis. Consistent with a mitotic role for cdr2, we observed that NCBI's conserved domain database [26] found significant (p = 5×10^−5^) homology between the cdr2 N-terminal coiled-coil/leucine zipper domain and the coiled-coil regions of the structural maintenance of chromosomes (SMC) family of proteins [27]. While the overall identity between the respective domains is not high, the similarity is and the homology to both prokaryotic and eukaryotic SMC proteins is well conserved in mammalian as well as avian, amphibian and teleost cdr2. To test whether cdr2 may be important in mitosis, we performed cdr2 knockdown utilizing a pool of 4 cdr2-specific siRNAs that reduced cdr2 mRNA or protein levels (Figs. 3A, 3B, S3) to less than 10% of control siRNA-treated HeLa cells.

To examine mitotic cells after cdr2 knockdown, we imaged mitotic spindles in these cells by immunofluorescence microscopy, using a α-tubulin antibody and DAPI to visualize mitotic cells. While cdr2 knockdown in HeLa cells did not lead to overt cell cycle arrest (Fig. S3), we observed an increase in the number of multipolar spindles (Fig. 3C). Quantitation of this data revealed that 21% of cdr2 knockdown cells showed aberrant multipolar spindles 48 hours after cdr2 siRNA transfection (Fig. 3D), compared to 11% of control cells. We note that our observation of low-level multipolar spindle formation in control siRNA-treated HeLa cells is consistent with previous reports [28], [29].

### cdr2 interacts with and regulates c-myc during mitosis

**Figure 4:**
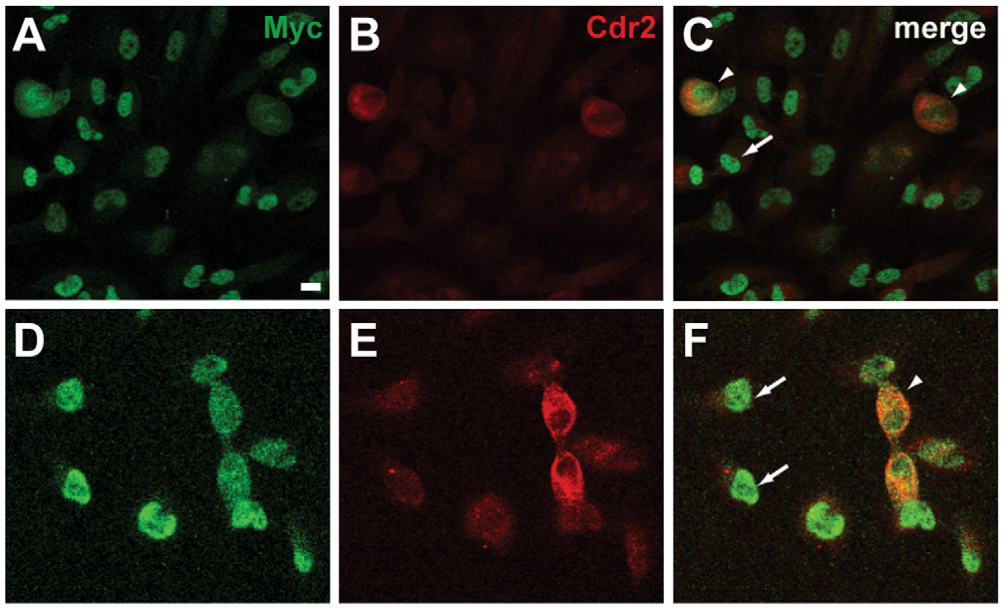
Cdr2 colocalizes with c-myc during mitosis. Low power (20X), thin section confocal images of HeLa cells stained with anti-c-myc (9402; A, D), anti-Cdr2 (4F5; B, E) and merged images (C, F). Arrows denote nuclear localized c-myc; arrowhead indicates mitotic cells with co-localized c-myc and cdr2. Scale bar = 20 µm.

**Figure 5:**
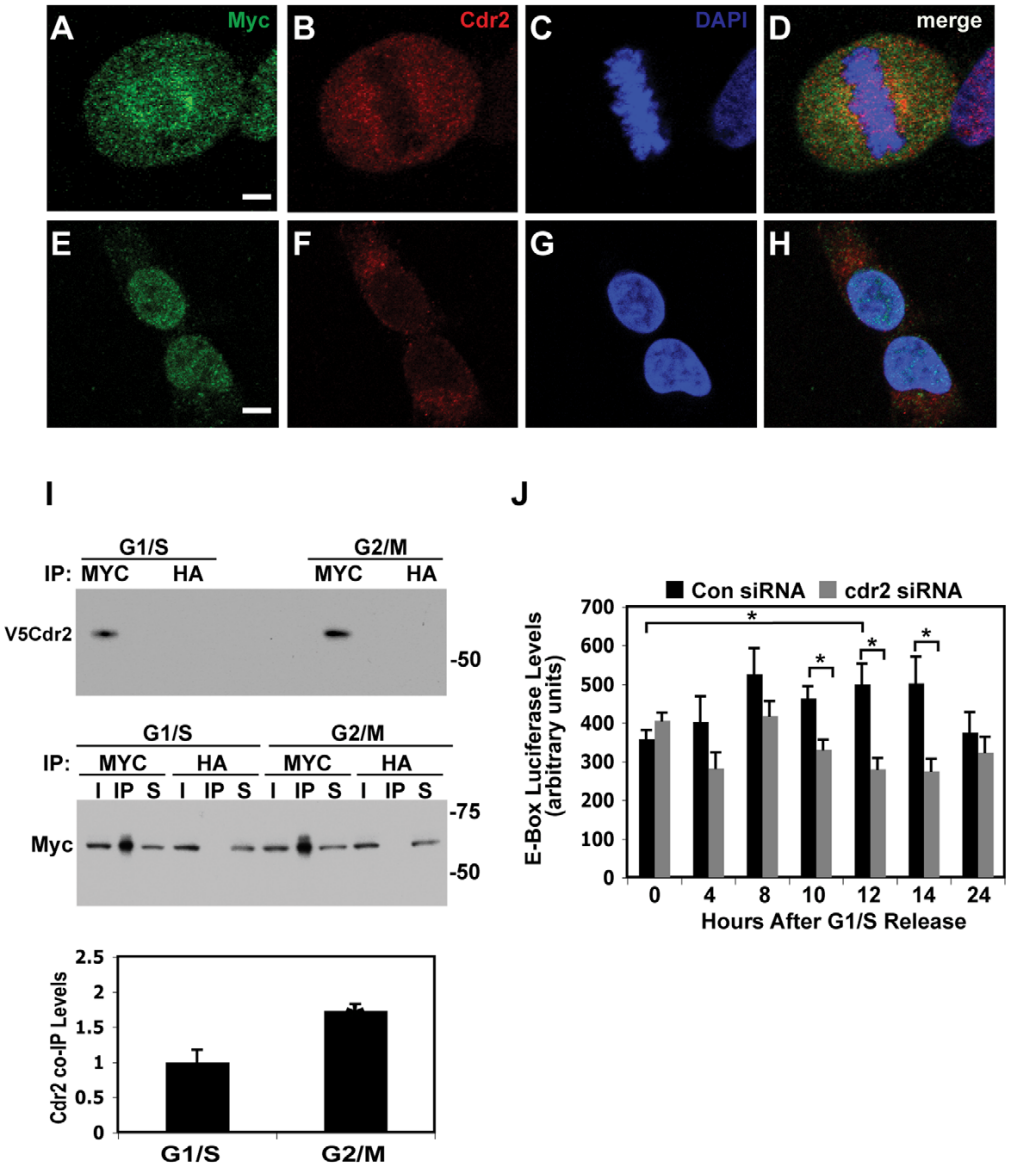
Cdr2 interacts with and regulates c-myc during mitosis. High power (63X), thin section confocal images of HeLa cells stained with anti-c-myc (9402; A, E), anti-Cdr2 (4F5; B, F), DAPI (C, G) and merged images (D, H); Scale bar = 20 µm. I. Immunoprecipitation of G1/S and G2/M-arrested V5cdr2 and c-myc transfected HeLa extracts with anti-c-myc (C3956) and control anti-HA, blotted with anti-V5 to detect co-immunoprecipitated V5cdr2. Middle, control anti-c-myc (9E10) western of above inputs, immunoprecipitates and supernatants. Bottom, quantitation of V5cdr2 co-precipitated by anti-c-myc, normalized to V5cdr2 input (data not shown); *p<0.01. J. Luciferase assay of G1/S released HeLa extracts transfected with c-myc responsive E-box luciferase and cdr2 or control siRNA pools; *p<0.05.

Previous studies [11] have shown that cdr2 interacts with c-myc *in vivo* in the cytoplasm of Purkinje neurons. Cdr2 overexpression can down-regulate c-myc-dependent transcription in tumor cells, although how the cytoplasmic cdr2 protein bound nuclear localized c-myc in this setting was unclear. The observation that cdr2 protein levels peak during mitosis suggested the possibility that it might gain access to c-myc during the breakdown of the nuclear envelope that occurs early in mitosis. To assess whether cdr2 and c-myc colocalize during mitosis, we performed confocal microscopy on HeLa cells. During interphase cdr2 levels were barely detectable and localized to the cytoplasm, while c-myc was localized exclusively to the nucleus (Fig. 4). In contrast during mitosis, cdr2 levels were significantly higher and was co-localized with c-myc protein that had redistributed away from DNA (Fig. 4). High power confocal analyses confirmed the cdr2-c-myc colocalization during mitosis (Fig 5A–H). While both cdr2 and c-myc displayed diffuse staining patterns that did not overlap with the DAPI signal, we noted that the bulk of the colocalized cdr2 and c-myc preferentially localized proximal to the spindle poles (Fig. 5A–D) consistent with prior observations made on c-myc alone [30]. Following completion of mitosis, c-myc re-localized to the nucleus while cdr2 localized to the cytoplasm (Fig. 5E-H). We previously demonstrated that cdr2 and c-myc co-immunoprecipitated in mouse brain extracts, as well as in tissue culture cells [11]. To test whether cdr2 and c-myc could physically interact during mitosis, we performed co-immunoprecipitations of HeLa cells transiently transfected with tagged cdr2 and c-myc expression constructs. After transfection, we synchronized HeLa cells in G1/S or G2/M and performed either c-myc or control immunoprecipitations followed by western blots to detect cdr2 protein. These experiments (Fig. 5I) demonstrated a direct interaction between cdr2 and c-myc is preferentially evident during G2/M-phase, as compared to G1/S, and support the conclusion that cdr2 binds to c-myc when c-myc is not in contact with DNA. The preferential interaction of cdr2 and c-myc during G2/M in this experiment does not appear to be due to elevated levels of endogenous cdr2 protein during G2/M since transfected cdr2 was present at similar levels during G1/S and G2/M in these experiments (data not shown).

### cdr2 regulates c-myc target genes in tumor cells

We previously demonstrated that cdr2 could inhibit c-myc-dependent transcription in overexpression experiments [11]. To test whether cdr2 could regulate c-myc target genes, we performed loss-of-function siRNA knockdown studies in HeLa cells. We first tested whether cdr2 knockdown could affect output from a c-myc responsive E-box-luciferase reporter. HeLa cells were transfected with the E-box-luciferase reporter, cdr2 siRNAs or control siRNAs, and an EGFP expression construct for normalization [11] followed by synchronization at the G1/S border. Following release from a G1/S block, we harvested cells and assayed luciferase activity, and we monitored the cell cycle profile by flow cytometry. We observed a significant spike in E-box-luciferase reporter activity 12 hours after release into S phase, which correlated with the peak of cells in late G2/M (Fig. 5J, black bars and data not shown), and with a prior report that c-myc-dependent transcription spikes in late G2 [31]. Further, we found that cdr2 knockdown led to a decrease in E-box-luciferase reporter activity that was maximal as cell passaged through mitosis (Fig. 5J). While the E-box luciferase reporter can also be responsive to transcription factors other than c-myc, this observation led us to further analyze cdr2 regulation of c-myc dependent transcription.

To assess whether cdr2 could regulate endogenously expressed c-myc target genes in HeLa cells, we compared the transcriptional profile of cells in the presence or absence of cdr2 using siRNA knockdown 3 hours after release from a mitotic block. Because there is a general shutdown of transcription during mitosis [32], these studies likely reflect a combination of cdr2-dependent changes in target gene expression at G2/M and M/G1 transitions. We identified 324 cdr2-regulated genes whose expression was changed by 1.5 fold or greater (Table S2). A gene ontology analysis of this data set revealed enrichment for genes involved in chromosomal, chromatin and nucleosome regulation as well as in cell cycle and mitotic biology (Table S1).

**Figure 6:**
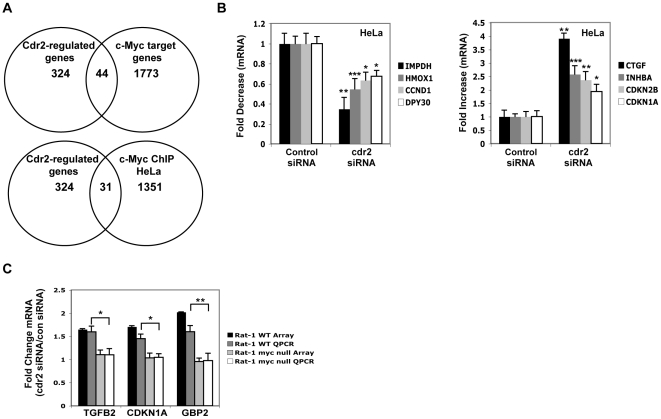
Cdr2 knockdown causes mis-regulation of endogenous c-myc target genes. A. Venn diagrams of microarray data of control and cdr2 siRNA-treated HeLa cells that recently exited mitosis. Upper, 44 genes overlap between 324 cdr2-regulated genes and 1773 c-myc target genes; lower, 32 genes overlap between cdr2-regulated genes and 1351 c-myc ChIP HeLa targets. B. qRT-PCR of select cdr2-regulated c-myc target genes that are decreased (left) and increased (right) following cdr2 knockdown (see Table 1); *p<0.05, **p<0.005, ***p<0.0001. C. Microarray and qRT-PCR analyses of three cdr2-regulated c-myc target mRNAs in wild type and c-myc null Rat-1 cells; *p<0.05, **p<0.005.

To assess whether cdr2 could affect c-myc target gene expression, we cross-referenced the 324 cdr2-regulated genes (Fig. 6A) with two different c-myc target gene lists, one a comprehensive list of c-myc target genes [33] (www.myccancergene.org) and the other a list of c-myc target genes identified by chromatin immunoprecipitation (ChIP) in HeLa cells [34]. This analysis generated two lists of putative cdr2-regulated c-myc target genes. A Fisher's exact test revealed that the observed degree of overlap was highly significant for both data sets; 44 genes overlapped (p = 7.4×10^−11^, odds ratio = 3.4) with the c-myc cancer gene database and 31 genes overlapped with the c-Myc ChIP list (p = 3.2×10^−7^, odds ratio = 3.0). We independently validated changes in the steady state levels of 30/44 (68%) of these transcripts from both lists by qRT-PCR (Fig. 6B; Table 1).

**Table 1:** Validated cdr2-regulated c-myc target genes.

	Gene	Array	qPCR	MYC DB	Myc ChIP
1	TGFBR2	1.7	5.59	Y	
2	COL3A1	1.8	5.02	Y	
3	CTGF	2.39	3.91	Y	
4	INHBA	3.7	2.58	Y	
5	CDKN2B	1.62	2.37	Y	
6	CDKN1A	1.63	1.93	Y	Y
7	SERPINE1	1.86	1.82	Y	
8	GBP2	1.76	1.7	Y	
9	TGFB2	1.5	1.54	Y	Y
10	SERPINE2	1.79	1.43	Y	
11	S100A2	1.61	1.43	Y	
12	IMPDH1	0.32	0.34	Y	Y
13	CDC25A	0.46	0.44	Y	Y
14	HMOX1	0.62	0.54	Y	
15	ERO1L	0.49	0.6	Y	
16	CXCL2	0.58	0.62	Y	
17	CCND1	0.56	0.63	Y	
18	ETS2	0.59	0.63	Y	Y
19	DPY30	0.57	0.67	Y	
20	MASA	0.59	0.69	Y	Y
21	PGK1	0.63	0.7	Y	Y
22	RBBP4	0.6	0.71	Y	Y
23	ASNS	0.61	0.72	Y	
24	CCNB1	0.62	0.74	Y	Y
25	DKC1	0.63	0.79	Y	
26	NUF2	0.55	0.41		Y
27	CENPA	0.55	0.42		Y
28	NDC80	0.45	0.63		Y
29	CDCA3	0.39	0.64		Y
30	KIF15	0.55	0.69		Y

The table lists 30 validated cdr2-regulated c-myc target genes. Cdr2 knockdown microarray and qRT-PCR data are indicated for each gene; values >1 indicate up-regulation, values <1 down-regulation. Y indicates whether the gene is present in the c-myc cancer gene or c-myc ChIP date sets.

To investigate how cdr2 might be regulating these transcripts, we focused on 15 well-studied genes which are known to be either activated or repressed by c-myc and which have been previously validated (in c-myc null cells, chromatin immunoprecipitation or nuclear run-on experiments; [33], [35]). For 13/15 (87%) of these transcripts, cdr2 acted as an agonist of c-myc function (Table 2). In roughly half of these cases, c-myc acts as a transcription inducer and in half a transcription inhibitor, consistent with known actions of c-myc to activate some genes via an E-box, and to repress others, for example, through actions on cell cycle genes through binding to INR sequences [36], [37]). Figure 6B shows examples of c-myc target mRNAs that are either down-regulated or up-regulated in the absence of cdr2.

**Table 2:** Well-studied, validated cdr2-regulated c-myc target genes.

	Gene	Myc Regulation	Array	qPCR	Cdr2 Regulation	Predicted cdr2 action on c-myc
1	HMOX1	D	0.62	0.54	U	inhibit
2	GBP2	D	1.76	1.7	D	activate
3	CDKN1A	D	1.63	1.93	D	activate
4	CDKN2B	D	1.62	2.37	D	activate
5	INHBA	D	3.7	2.58	D	activate
6	CTGF	D	2.39	3.91	D	activate
7	TGFBR2	D	1.7	5.59	D	activate
8	IMPDH1	U	0.32	0.34	U	activate
9	CCND1	U	0.56	0.63	U	activate
10	DPY30	U	0.57	0.67	U	activate
11	PGK1	U	0.63	0.7	U	activate
12	ASNS	U	0.61	0.72	U	activate
13	CCNB1	U	0.62	0.74	U	activate
14	DKC1	U	0.63	0.79	U	activate
15	S100A2	U	1.61	1.43	D	inhibit

The table lists 15 genes that are either up-regulated (U) or down-regulated (D) by c-myc and have been previously independently validated. For 13/15 of these mRNAs (#'s 2-14), cdr2 appears to activate c-myc function, while for 2/15 genes (#'s 1 and 15) cdr2 appears to inhibit c-myc function.

To evaluate whether cdr2 action on c-myc target genes was directly dependent on c-myc, we examined the effect of cdr2 knockdown in a Rat-1 c-myc null cell line [38]. We undertook microarray experiments in wild type Rat-1 cells and c-myc-null cells exiting mitosis that were treated with cdr2 or control siRNA pools (Fig. S4). We identified four genes (CDKN1A, GBP2, INHBA, TGFB2) of the 30 validated cdr2-regulated c-myc target genes in HeLa cells that were also represented on the rat microarray. Microarray and qRT-PCR studies revealed that three of these genes, CDKN1A, GBP2 and TGFB2, are regulated by cdr2 in wild type Rat-1 cells but not in c-myc null cells (Fig. 6C), suggesting that these three genes require c-myc to show cdr2 effects on gene regulation. INHBA was not found to be cdr2-regulated in wild type Rat-1 cells.

Several validated cdr2-regulated c-myc target genes (CCNB1, CDCA3, CENPA, KIF15, NDC80 and NUF2) are mitosis-related mRNA's (Table 3) that are also down-regulated in the absence of cdr2. Moreover, four of these genes (CENPA, KIF15, NDC80 and NUF2) are involved in kinetochore and spindle biology (reviewed in [39]). Additionally, cdr2 knockdown lead to a decrease (Table 3) in four other transcripts (AURKA, CENPE, SPC25 and TTK), which are involved in kinetochore and spindle biology, but are not known to be c-myc targets genes ([40] and reviewed in [41]). Although our data do not rule out the possibility that cdr2 could have some indirect actions on c-myc target genes, collectively, the data strongly support a model (see Discussion) whereby cdr2 interacts with c-myc during mitosis, but not during other stages of the cell cycle, to promote downstream actions of c-myc on transcriptional targets that mediate effects on mitotic spindle assembly and mitotic passage.

**Table 3:** Cdr2-regulated mitotic genes.

Gene	Array	qPCR	c-myc ChIP (HeLa)	Kinetochore	Spindle
NUF2	0.55	0.41	Y	Y	
CENPA	0.55	0.42	Y	Y	
NDC80	0.45	0.63	Y	Y	
CDCA3	0.39	0.64	Y		
KIF15	0.55	0.69	Y		Y
CCNB1	0.61	0.73	Y		
CENPE	0.46	0.44			Y
SPC25	0.62	0.54		Y	
TTK	0.59	0.63			Y
AURKA	0.63	0.78			Y

List of 10 mitosis-related mRNAs from the 324 cdr2-regulated genes data set. Cdr2 knockdown microarray and qRT-PCR data are indicated for each gene; values <1 indicate down-regulation. Y indicates whether the gene is on the c-myc ChIP list and whether it is involved in kinetochore or spindle function.

### Cdr2 regulates cellular proliferation

Our findings of a functional interaction between cdr2 and c-myc in mitosis, together with the fact that cdr2 is expressed in gynecologic [4] and renal [42] tumors raised the question whether cdr2 can promote tumor growth. Remarkably, several validated cdr2-regulated c-myc target genes in HeLa cells have roles in cell cycle biology (Table 1, Fig. 6B). Specifically, of the c-myc target genes that are increased following cdr2 knockdown, several are known to inhibit the cell-cycle (CDKN1A, CDKN2B, and TGFBR2; Table 2 and [33]). These genes are normally repressed by c-myc, as c-myc is promoting the cell cycle and driving proliferation. In contrast, some cell cycle-promoting genes such as CCNB1 and CCND1, which are known to be up-regulated by c-myc, are down-regulated following cdr2 knockdown (Table 2). Taken together, the data form a coherent hypothesis—that cdr2 action on c-myc leads to a concerted action on target genes to promote cellular proliferation, and that this action is inhibited by cdr2 knockdown.

**Figure 7:**
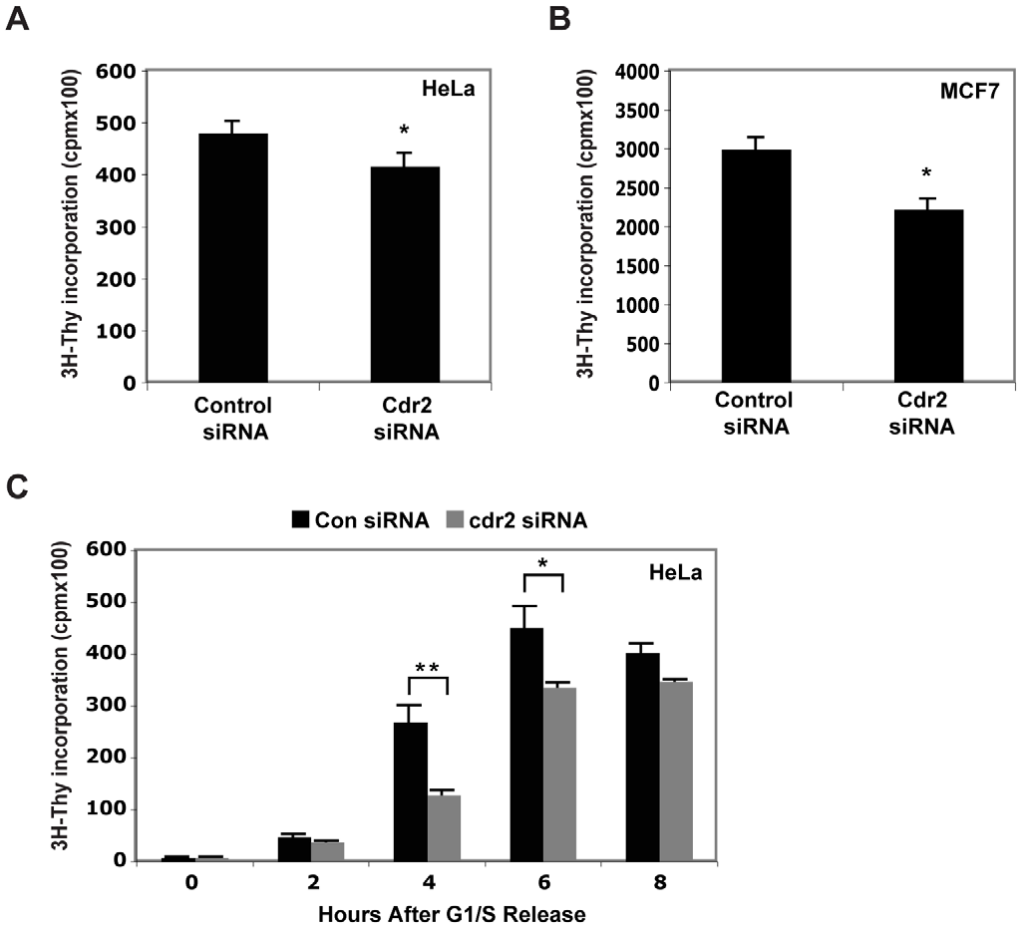
Cdr2 knockdown causes impaired proliferation. Steady state proliferation (^3^H-thymidine incorporation (cpm)) of HeLa (A) and MCF7 (B) cells after control or cdr2 siRNA treatment. *p<0.05. C. HeLa ^3^H-thymidine incorporation (cpm) time course in cells released from G1/S block; *p<0.05, **p<0.005.

This hypothesis prompted us to ask whether cdr2 could regulate cellular proliferation. Using ^3^H-thymidine incorporation to assay steady state proliferation, we found that cdr2 knockdown led to a 14% (p<0.05) decrease in HeLa cell proliferation (Fig. 7A). We obtained similar results (25% reduction, p<0.05) in the MCF7 breast cancer cell line (Fig. 7B), which also expresses cdr2 [10]. In addition, cdr2 knockdown HeLa cells exhibit delayed kinetics entering into S phase following a G1/S arrest (Fig. 7C), with the largest reduction in proliferation observed four hours following release.

**Figure 8:**
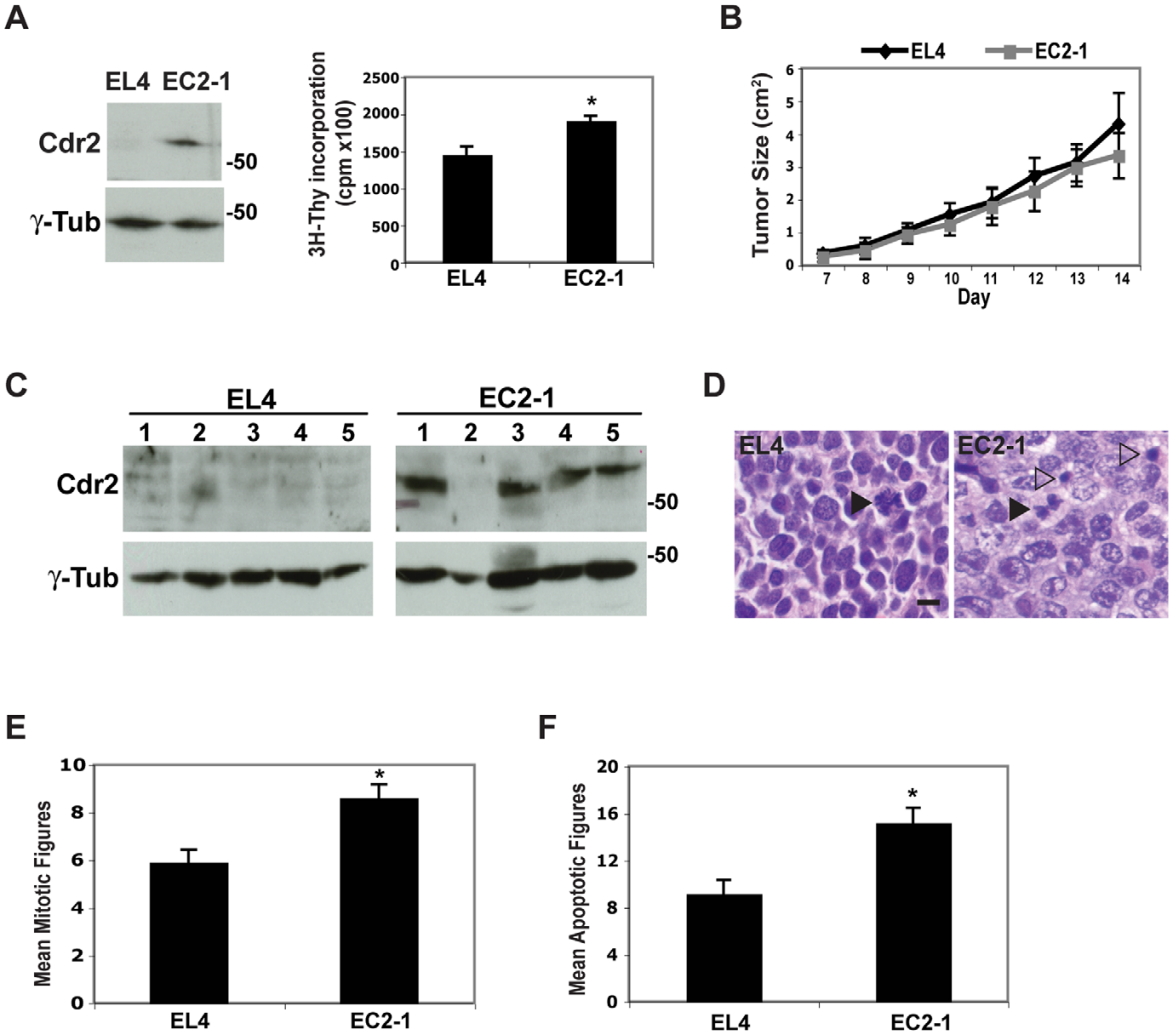
Cdr2 overexpression in tumors drives the cell cycle. A. Western blots of EL4 and EC2-1 extracts probed with cdr2 and γ-tubulin antisera (left) and steady state proliferation (^3^H-thymidine incorporation (cpm)) of EL4 or EC2-1 cells lines (right); *p<0.05. B. Quantitation of tumor size (cm^2^) in EL4- (black) and EC2-1- (gray) injected nude mice at indicated days. C. Western blots of tumor extracts from mice injected with EL4 or EC2-1 cells probed with cdr2 and γ-tubulin antisera. D. Representative hematoxylin and eosin stains of EL4 (left) or EC2-1 (right) tumor sections (60X). Black arrowheads indicate mitotic figures; clear arrowheads indicate apoptotic figures. Scale bar = 10 µm. E. Quantitation of mitotic figures. *p<0.05. F. Quantitation of apoptotic figures; *p<0.05.

Because cells lacking cdr2 exhibit impaired proliferation, we tested whether cdr2 overexpression could promote cell cycle progression in tumors. We transfected T7cdr2 into EL4 lymphoblastoid cells that have undetectable cdr2 protein levels [10] in order to generate a stable T7cdr2-expressing clonal cell line (EC2-1), (Fig. 8A). When compared to EL4 cells, EC2-1 cells exhibited 1.3-fold higher steady state ^3^H-thymidine incorporation (Fig. 8B) suggesting that T7cdr2 overexpression may drive the cell cycle. We obtained similar results in both NIH3T3 cells stably over-expressing T7cdr2 and cerebellar granule cell neurons transiently over-expressing T7cdr2 (Fig. S5).

To evaluate whether cdr2 overexpression has growth-promoting activity *in vivo*, we injected parent EL4 and EC2-1 cells into the flanks of nude mice and monitored tumor growth over the course of two weeks. Importantly, cdr2 expression was maintained in 4 out of the five EC2-1 tumors (Fig. 8C) for up to two weeks in the absence of any drug selection. While we did not observe any significant differences in tumor growth rate nor in tumor size (Fig. 8B) before animals were sacrificed, the cdr2 positive EC2-1 derived tumors exhibited a significant increase in the number of mitotic figures compared to that of the EL4 cell-derived tumors (Fig. 8F). This result is consistent with the elevated proliferation observed *in vitro* with EC2-1 cells. We also assessed the degree of apoptotic death in these cells, since cells may respond to c-myc over-activation by undergoing programmed cell death [43]. Consistent with our observation that the predominant action of cdr2 was to promote c-myc activity, we observed an increase in the incidence of apoptotic figures (Fig. 8G) in the EC2-1 tumors relative to EL4 tumors. While we did not observe that cdr2 promoted c-myc-dependent transcription of apoptotic genes, these data may provide a plausible explanation for why cdr2 overexpression in EC2 cells led to an increase in cell cycling yet EC2-1 tumors were not larger than the control EL4 tumors.

## Discussion

We have found that the paraneoplastic cerebellar degeneration related antigen cdr2 acts as a mitotic protein in tumor cells. Normally cdr2 expression is mostly restricted within the brain to post-mitotic Purkinje neurons [5], [11], raising the question of whether its role in mitosis is a novel function of the protein or a reflection of its normal neuronal biology. Many links have been made between the cell cycle machinery and neuronal biology since the discovery of oncogenes—for example, the ras [44] and myc proteins [11], [45] are expressed in Purkinje neurons, and the cell cycle protein cdk5 is involved in dendrite formation [46] Thus the evolving idea that cell cycle pathways are utilized for parallel pathways in dividing cells and neurons is supported by the finding here that the Purkinje protein cdr2 functions in mitotic control in tumor cells.

Cdr2 levels are regulated in mitosis both by *de novo* expression of the transcript and protein (Fig. 1), and through down-regulation at least in part by APC/C-mediated ubiquitination and proteasome degradation. Cdr2 levels are high in brain, raising the question of whether cdr2 is regulated in a similar manner in neurons. The APC/C is active in brain extracts and components of the APC/C (APC2, Cdc27 and Cdh1), are believed to be expressed solely in the nucleus in the cerebellum [47], while cdr2 is cytoplasmic in Purkinje neurons, suggesting that cdr2 may escape APC/C-mediated ubiquitination. However, recent papers have ascribed axonal and dendritic roles to Cdh1 [48] and Cdc20 [49], respectively, which could have relevance to cdr2, which is present in the neuronal cytoplasm and proximal dendrites [5], [11]. More generally, the role we observe for cdr2 in mitosis and spindle formation in dividing cells may reflect a role for cdr2 in Purkinje neuronal dendritic remodeling, as such a role has been proposed for the APC/C^Cdc20^ in neurons [49].

Our results also suggest specific consequences for cdr2 mitotic expression in gynecologic cancers, and thereby address the paradox of why these tumors express the cdr2 antigen despite the fact that this allows them to be targeted by the immune system [9]. Mechanistically, APC/C dysfunction in these tumors may play a role; such dysfunction has been proposed to be a hallmark of malignant tumors, as mitotic APC/C substrates were significantly up-regulated in malignant versus non-malignant human cancers in a survey of more than 1600 benign and malignant tumors [50].

Interestingly, we note that human cdr2 is reported to harbor cell cycle-regulated phosphorylation sites (/PhosphoSitePlus, www.phosphosite.org/ [51]) adjacent to one of its destruction boxes. These sites, serines 309–311, are immediately C-terminal to the second destruction box (RKPL, 303–306aa, Fig. 2), and two of these sites, serines 309 and 311, are phosphorylated in HeLa cells during G1 but not during mitosis [51]. Together with the data presented here and with the proximity of these sites to the destruction box, we suggest a model whereby cdr2 phosphorylation at serines 309–311 might lead to enhanced APC/C-mediated ubiquitination and subsequent degradation during G1, but not during mitosis when these sites are un-phosphorylated and when cdr2 levels are at their peak. Consistent with this model, our previous observation that c-myc preferentially co-immunoprecipitated *in vivo* with a faster-migrating and possibly unphosphorylated cdr2 species [11] suggests that the cdr2:c-myc interaction may be favored when cdr2 is un-phosphorylated during mitosis.

**Figure 9:**
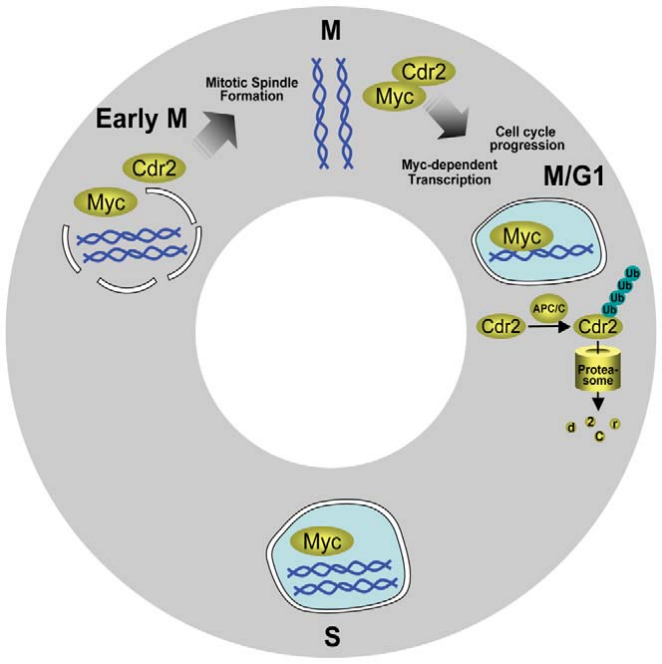
Model of cdr2 action in mitosis. Cdr2 levels increase in G2, peak in mitosis and decrease as cells exit mitosis. Nuclear envelope breakdown in prometaphase allows newly synthesized cdr2 access to c-myc protein, which, like other transcription factors [61], partitions itself off DNA during chromatin condensation, coinciding with mitotic shutdown of transcription. Cdr2 colocalization with c-myc is first apparent during metaphase (Figs. 4C, 5D) and peaks during telophase/cytokinesis (Fig. 4F) after which c-myc relocalizes to the nucleus (Figs. 4D, 5E). During late mitosis/early G1, cdr2 KEN and D boxes are recognized by the APC/C, which ubiquitinates cdr2, targeting it for degradation by the proteasome. Cdr2 knockdown cells show defects in mitotic spindle formation, cell cycle progression and c-myc-dependent transcription. Based on our current results, and the observations that cdr2 is preferentially phosphorylated during G1 but not mitosis [51], and that a smaller migrating, possibly dephosphorylated, isoform of cdr2 interacts with c-myc *in vivo* [11], we hypothesize that unphosphorylated cdr2 interacts with c-myc to prevent c-myc degradation, thereby allowing subsequent c-myc-dependent transcription of genes to occur upon resumption of transcription following mitosis. At this point phosphorylation of cdr2 leads to recognition by the APC/C, targeting it for degradation by the proteasome. In tissue culture, overexpression of cdr2 may lead to several effects, including aberrant sequestration of c-myc and inhibition of c-myc activity [11]. Taken together, the data suggest an overall hypothesis, in which that cdr2 action on c-myc leads to a concerted action on target genes to promote cellular proliferation, and that this action is inhibited by cdr2 knockdown. Disruption of cdr2:c-myc control may therefore disrupt mitotic exit and impact the growth of cdr2-expressing tumors.

We find that inappropriate expression of cdr2 in tumor cells can impact c-myc activity. The cdr2-c-myc interaction is specific and mediated by the cdr2 leucine zipper/coiled-coil domains [11]. Further, we find that this interaction occurs during mitosis (Figs. 4, 5) and in Rat-1 cells cdr2 acts synergistically with c-myc to regulate endogenous c-myc target genes (Fig. 6C). And while CDKN1A's effects in HeLa cells would be expected to be at least partially blocked by the HPV protein E7 [52], we note that the set of cdr2-regulated c-myc target genes in both Rat-1 and HeLa cells (Figs. 6B, 6C, Table 1), including the mitosis-related mRNAs (Table 3), encode proteins important for cell cycle progression. Also, given the recent finding that c-myc controls the expression of two mitotic spindle-associated proteins in breast tumor cell lines [53], our data that cdr2 may impinge on c-myc regulation of other mitosis-related transcripts during the G2/M transition in HeLa cells is consistent with a role for c-myc during G2 [31]. While further experiments are needed to clarify cdr2 function in spindle biology and whether those effects are independent of or connected to cdr2 regulation of c-myc-dependent transcription, these observations suggest a model (Fig. 9) in which cdr2 acts during mitosis, in part through interactions with c-myc, to regulate a cascade of actions related to tumor cell growth.

These findings have several implications for gynecologic cancer. Cdr2 is not expressed at high levels outside of the brain and testis, but is expressed in 60% of ovarian and 22% of breast tumors and in many transformed cell lines [4], [10]. Our data suggests that cdr2 may contribute to tumorigenesis, since cdr2 expression levels correlate with cell proliferation. Although we do not see enhanced tumor growth mediated by cdr2 in EL4 tumor cells, a random screen for focus-forming genes in NIH3T3 cells [54] categorized cdr2 as “moderately” oncogenic. It may be that the oncogenic potential of cdr2 can be offset by the ability to trigger apoptotic death, as has been noted for c-myc [43]. Our findings of a function for cdr2 in tumor cell mitosis, together with its normally restricted tissue expression, suggest that it may serve as a tumor target with potential for a high therapeutic index of action.

## Materials and Methods

### Plasmids

We used the Rapid DNA Ligation Kit (Roche) for ligations. Point mutations were made with the QuikChange Kit (Stratagene). Primers (Table S3) were synthesized by Operon. Mark Kirschner (Harvard) provided the HACdc20, HACdh1 and CyclinB1 vectors; Dirk Bohmann (U. of Rochester) provided the HAUbiquitin construct; and Mary Hatten (Rockefeller University) provided the pCXiBSR vector.

### RNA, RT-qPCR

RNA was extracted using the RNeasy Kit (Qiagen). RT-qPCR was performed as described previously [55]. PCR conditions were determined independently for each primer pair using a temperature gradient followed by melting curve and we also calculated the amplification efficiency for all primer pairs. The relative amount of initial mRNA copies was determined using the Pfaffl method [56].

### Cell culture, synchronization, transfections

All cell lines were maintained at 37°C/5% CO_2_ in DMEM (Mediatech), 10% fetal bovine serum (Gemini) and pen-strep (50 U/ml; Invitrogen). Rat-1 TGR (wild type) and Rat-1 15.19 (c-myc null) cells were a gift from Michael Cole (Dartmouth). Cerebellar granule neurons were cultured according to established protocols [57]. We synchronized cells by sequential thymidine/aphidicolin block [14]. For mitotic arrest, thymidine/aphidicolin were released in media with 0.1 µg/ml demecolcine or 0.8 µM nocodazole. For mitotic release, nocodazole-treated cells were washed and allowed to grow as indicated. Half of the cells were used for flow cytometry and half for Western or RNA analysis. Nocodazole, demecolcine, thymidine, aphidicolin and cycloheximide were from Sigma. Transfections were performed using Fugene6 (Roche) or Lipofectamine2000 (Invitrogen).

siRNA experiments were done using non-targeting control, and human or rat cdr2 siRNA pools (Dharmacon). Pilot experiments demonstrated that potential off-target effects observed with high concentrations of single siRNAs were not seen when we used siRNA pools at low nanomolar siRNA concentrations (data not shown). 40 µM siRNA stocks were diluted in OptiMEM and Lipofectamine2000 and were added to cells at a 10 nM final concentration. For microarrays, cells were treated with cdr2 or control siRNAs 48 hours prior to synchronization in mitosis. We harvested RNA 3 hours after release from a mitotic block. Luciferase assays were done using a luciferase assay kit (Promega). Activity was calculated by normalizing raw firefly luciferase activity to GFP expression levels measured by western blot. For proliferation assays, cells were plated in 96-well plates, labeled for 6 hours with ^3^H-thymidine (GE Healthcare) and harvested on a Unifilter96 GF/C filter and counted on a TopCount machine (PerkinElmer). Cycloheximide stocks were diluted to 35 µg/ml for protein stability studies.

### Viral transduction

T7cdr2 cDNA was cloned into pCXiBSR. pCXiBSR alone and pCX-T7cdr2iBSR were transfected into HEK293 to produce retroviruses [58]. Supernatants were used to transduce NIH3T3 cells; transduced cells were selected with 20 µg/ml blasticidin (Invitrogen), which killed all non-infected cells. Blasticidin-resistant cells were expanded and T7cdr2 expression was confirmed by western blot and immunocytochemistry.

### Flow Cytometry

We stained cells with TOPRO3 (Invitrogen) to measure DNA content. Cells were harvested by trypsinization, rinsed and re-suspended in PBS/2 mM EDTA, fixed by addition of cold 70% ethanol. Cells were spun at 1000 rpm for 5 minutes, 4°C and then re-suspended in 0.5 ml staining solution (950 µl PBS/2 mM EDTA, 20 µl RNaseA (10 mg/ml; Clontech), TOPRO3 (0.4 µM)). Reactions were incubated at 37°C for 30 minutes then run on a FACSCalibur machine (BD Biosciences). Analysis was performed with Cell Quest (BD Biosciences) and FlowJo software.

### Microarray

RNA for microarrays was harvested using the RNeasy kit. RT, second strand synthesis, cDNA purification and *in vitro* transcription with Biotin-11-UTP were performed using the MessageAmp II aRNA Amplification Kit (Ambion). Hybridization of the aRNA to Affymetrix Human Genome U133 Plus 2.0 Arrays was carried out by the Rockefeller University Genomics Resource Center. Data was analyzed using GenespringGX software version 7.3.1 (Agilent). We performed 1-way ANOVA without multiple testing correction, p<0.01 to identify transcripts 1.5-fold differentially regulated between control and cdr2 siRNA treated cells in biological triplicate and eliminated transcripts below raw expression levels of 100 (arbitrary units). Microarray data (GSE20037) has been submitted to the Gene Expression Omnibus.

### Antibodies

Anti-cdr2/3 (Yo) antibodies are derived from PCD patient sera and CSF. Cdr2 monoclonal (4F5) is from Abnova. Cdr2 rabbit polyclonal is from Novus (NB110-58345). CyclinB1 (C8831), γ-tubulin (GTU88), HA (H6908), and c-myc (9E10 (mouse) and C3956 (rabbit)) antibodies are from Sigma. α-tubulin (DM1A), APC11, Cdc23 and APC1 antibodies are from Abcam. Anti-T7 is from Novagen. Horseradish peroxidase (HRP)-conjugated Anti-HA (3F10) is from Roche. Anti-Cdc27 (AF3.1) was a gift from Julian Gannon (Clare Hall Labs, Cancer Research UK). c-myc antibody (9402) is from Cell Signaling Technology. Anti-GFP (JL8) is from Clontech. Mouse, rabbit and human HRP- and FITC/Cy5-conjugated antibodies are from Jackson. Mouse, rabbit and human Alexa488/594/647-conjugated antibodies are from Invitrogen.

### Western blot and Immunocytochemistry

Western blots were performed as previously described [59], [60] and were quantitated using a Versadoc imaging system (BioRad).

Cells were plated on collagen-coated slides (BD Biosciences). Cells were rinsed in PBS, fixed with 3.7% paraformaldehyde in PBS (pH 7.4) for 15 minutes; permeabilized in 0.5% NP-40/PBS for 20 minutes; blocked in 0.2% gelatin/0.5% bovine serum albumin/PBS for 20 minutes, all at room temperature. Antibodies were diluted in block buffer and incubated overnight at 4°C. Fluorescent secondary antibodies were diluted in block buffer and incubated for 30 minutes at room temperature. Cells were rinsed with PBS. DAPI (4′,6-Diamidino-2-phenylindole dihydrochloride; Sigma) was included in the final wash before cover-slipping with GelMount (Biomeda). Slides were viewed on a Zeiss Axioplan microscope. Images were captured with a Hamamatsu Digital Camera. Image processing was performed using AxioVision 4.4 (Zeiss). Confocal laser scanning microscopy images were collected at the Bio-Imaging Resource Center at Rockefeller University with a Zeiss Axiovert200 microscope and LSM510 META version 3.2 software.

We identified mitotic spindles using DAPI and α-tubulin immunofluorescence. Prior to fixation in 4% formaldehyde (pH 6.9) with 100 mM K-PIPES, 1 mM MgCl2, 0.1 mM CaCl2 and 0.1% Triton X-100, cells were incubated at 37°C in microtubule stabilizing buffer (4M glycerol, 100 mM K-PIPES (pH 6.9), 1 mM EGTA, 5 mM MgCl_2_, 0.5% Triton X-100). >750 cells were counted for each condition.

### *In vitro* ubiquitination and degradation assay

^35^S-Met (GE Healthcare)-T7cdr2 was synthesized using a rabbit reticulocyte lysate (Promega). Human E1, UbcH5b (E2), ubiquitin and ubiquitin aldehyde are from Boston Biochem. The ubiquitination reaction consisted of ^35^S-T7cdr2, APC/C, HACdc20 or HACdh1, and 500 nM E1, 1.5 µM UbcH5b, ATP buffer (0.4 mM ATP pH 7.4, 3 mM creatine phosphate, 40 µM EDTA, pH 7.7), 15 µg ubiquitin, 2 µM ubiquitin aldehyde and was incubated at 30°C for 1 hour with aliquots taken at 0, 20 and 60 minutes and placed in Laemmli sample buffer (BioRad) and boiled for 4 minutes.

The APC/C was immunopurified from HEK293 cells released for 4 hours from a mitotic block. Cells were harvested in Swelling Buffer (20 mM HEPES, pH 7.5, 5 mM MgCl_2_ 5 mM KCl, ATP buffer, and a protease inhibitor cocktail (Calbiochem)). Cells were rapidly freeze-thawed in ethanol/dry ice and a 37°C water bath before passing through an ice-cold 18-gauge needle. Cells were spun at 4°C, 5000 rpm for 5 minutes, supernatants were collected and re-spun at 4°C, 14,000 rpm for 25 minutes. Supernatants were used as input for the APC/C (anti-Cdc27) immunoprecipitation. Following a 3 hour incubation at 4°C, Protein A beads (Sigma) were added for 1 hour. IP's were washed with swelling buffer/1% TritonX-100 and with swelling buffer. Purified APC/C was added on beads to the ubiquitination reaction. HACdc20 and HACdh1 were expressed in and purified from HEK293 cells using an HA antibody. Immunoprecipitated HACdc20 or HACdh1 were added on beads to the ubiquitination reaction. ^35^S-T7cdr2 was immunoprecipitated from rabbit reticulocyte lysates with anti-T7 and was added on beads to the ubiquitination reaction.

^35^S-labeled-T7cdr2, T7cdr2KEND1/D2 and cyclinB1 were synthesized *in vitro* using rabbit reticulocyte lysates and incubated with mitotic release HEK293 extracts which were prepared as described for the *in vitro* ubiquitination reaction. Reaction conditions were the same as for the ubiquitination reaction.

### Nude Mice Tumor Experiments

Stable cdr2-expressing EL4 cells (EC2) were derived following transfection of a cdr2 expression vector with G418 resistance and selection in G418. Cells were harvested, rinsed and counted with a hemocytometer before re-suspension at 10^7^ cells/ml. 10^6^ cells (100 µl) were injected into the left flank of 5 athymic nude mice (NU/J (Jackson Labs)) for each line. 5 control mice were injected with DMEM alone. Prior to injection, mice were anaesthetized with isofluorane (Baxter). Tumor size was measured daily (length and width) from the first sign of tumor growth. Animals were euthanized at two weeks post-injection or sooner if tumor size exceeded 5 cm^2^. Tissue was processed for western blot and histopathology, which was performed by the Research Animal Resource Center at Cornell University. Mitotic and apoptotic figures were quantitated by counting their total numbers per 60X section, 3 sections for each animal for a total of 10 animals with the counter blind to the cell line.

### Ethics Statement

All animals were handled in accordance with animal husbandry guidelines established and reviewed by the Rockefeller University Institutional Animal Care and Use Committee (IACUC), which comply with federal and state regulations that concern the use of experimental animals.

## Supporting Information

Table S1 The table lists GO term enrichment from the cdr2-regulated gene data set in HeLa cells exiting mitosis.
(0.02 MB XLS)

Table S2 Cdr2-regulated gene list. Genes are categorized by Affymetrix ID, fold change (value <1 indicates down-regulation; value >1, up-regulation) and gene description. The first list (Sheet 1) is of all 390 cdr2-regulated Affymetrix ID genes, the second list (Sheet 2) is 324 Affymetrix IDs and is the same as the first list except duplicate genes have been removed.
(0.10 MB XLS)

Table S3 This table lists all the primers used in this paper.
(0.04 MB XLS)

Figure S1 A. Cdr2 and cyclinB1 mRNA levels in HeLa cells at 0, 4, 8 and 12 hours after G1/S release, measured by qRT-PCR (normalized to b-actin mRNA). *p<0.01. B. Top, cdr2 (NB110) and a-tubulin western blots of cycloheximide-treated HeLa cells at indicated times; bottom, quantitation of cdr2 and tubulin levels at indicated times. C. Anti-T7 western blot of input and T7 or control (GFP) immunoprecipitations of T7cdr2-transfected HEK293 treated with DMSO or MG132 for six hours.
(0.99 MB TIF)

Figure S2 A. Anti-T7 western of input and T7 immunoprecipitates from T7cdr2 (top) and T7cdr2 KEN/D1D2 (bottom) transfected HEK293s. B. Anti-cdc27 immunoprecipitation and western blot with cdc27, cdc23 and APC11 antisera of HEK293s exiting mitosis. C. APC/C in vitro ubiquitination of 35S-cyclinB1 and 35S-Nova1. D. Anti-HA western of HA immunoprecipitation and inputs of HACdc20 and HACdh1 expressing HEK293s.
(0.87 MB TIF)

Figure S3 A. Western blot of HeLa extracts transfected with control and cdr2 siRNA pools probed with anti-cdr2 (4F5) and anti-γ-tubulin, showing triplicate experiments. ns indicates non-specific immunoreactive band detected by anti-cdr2 (4F5). B. Flow cytometry of G1S-arrested (top) and G1S-released (12 hour release, bottom) HeLa cells treated with control (left) or cdr2 siRNA pools (right) and stained with TOPRO3 to measure DNA content.
(0.60 MB TIF)

Figure S4 A. qRT-PCR of cdr2 mRNA levels (normalized to GAPDH mRNA) in Rat-1 wild type (left) and Rat-1 c-myc null cells (right) treated with control or rat cdr2 siRNA pools.
(0.16 MB TIF)

Figure S5 A. NIH3T3 steady state proliferation (3H-thymidine incorporation (cpm)) in stable cell lines expressing control (empty vector) or T7cdr2, *p<0.0001. B. Primary cerebellar granule cell neuron (GCN; 2DIV) steady state proliferation (3H-thymidine incorporation (cpm)) in cells transduced with control or T7cdr2 expressing virus. *p<0.005.
(0.36 MB TIF)
