# Supplementary figures and images for: The Onconeural Antigen cdr2 Is a Novel APC/C Target that Acts in Mitosis to Regulate C-Myc Target Genes in Mammalian Tumor Cells
Source: PLoS One. 2010 Apr 7;5(4):e10045. doi: 10.1371/journal.pone.0010045 (PMC2850929; doi:10.1371/journal.pone.0010045)

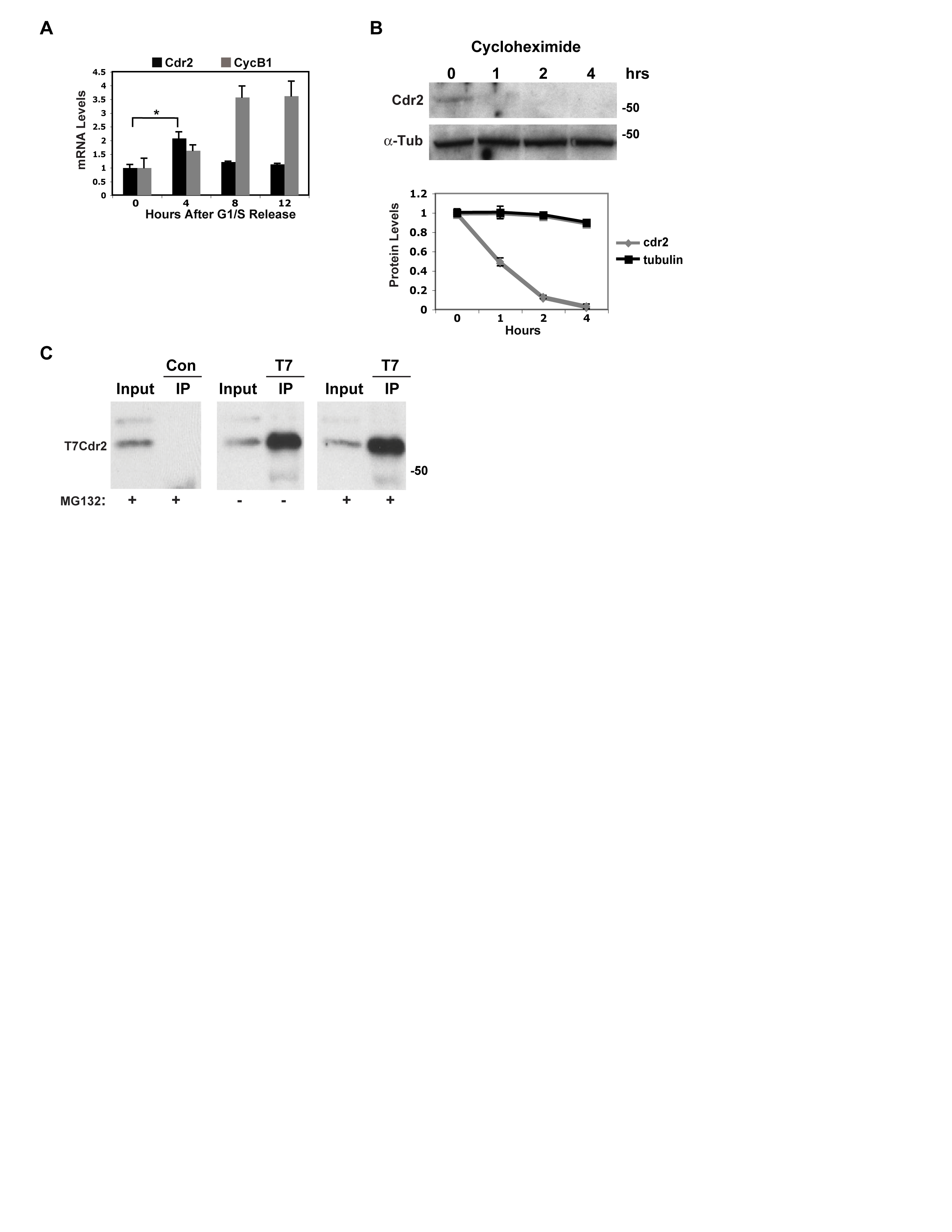

Supplement: Figure S1 — A. Cdr2 and cyclinB1 mRNA levels in HeLa cells at 0, 4, 8 and 12 hours after G1/S release, measured by qRT-PCR (normalized to b-actin mRNA). *p<0.01. B. Top, cdr2 (NB110) and a-tubulin western blots of cycloheximide-treated HeLa cells at indicated times; bottom, quantitation of cdr2 and tubulin levels at indicated times. C. Anti-T7 western blot of input and T7 or control (GFP) immunoprecipitations of T7cdr2-transfected HEK293 treated with DMSO or MG132 for six hours. (0.99 MB TIF) [file pone.0010045.s004.tif]

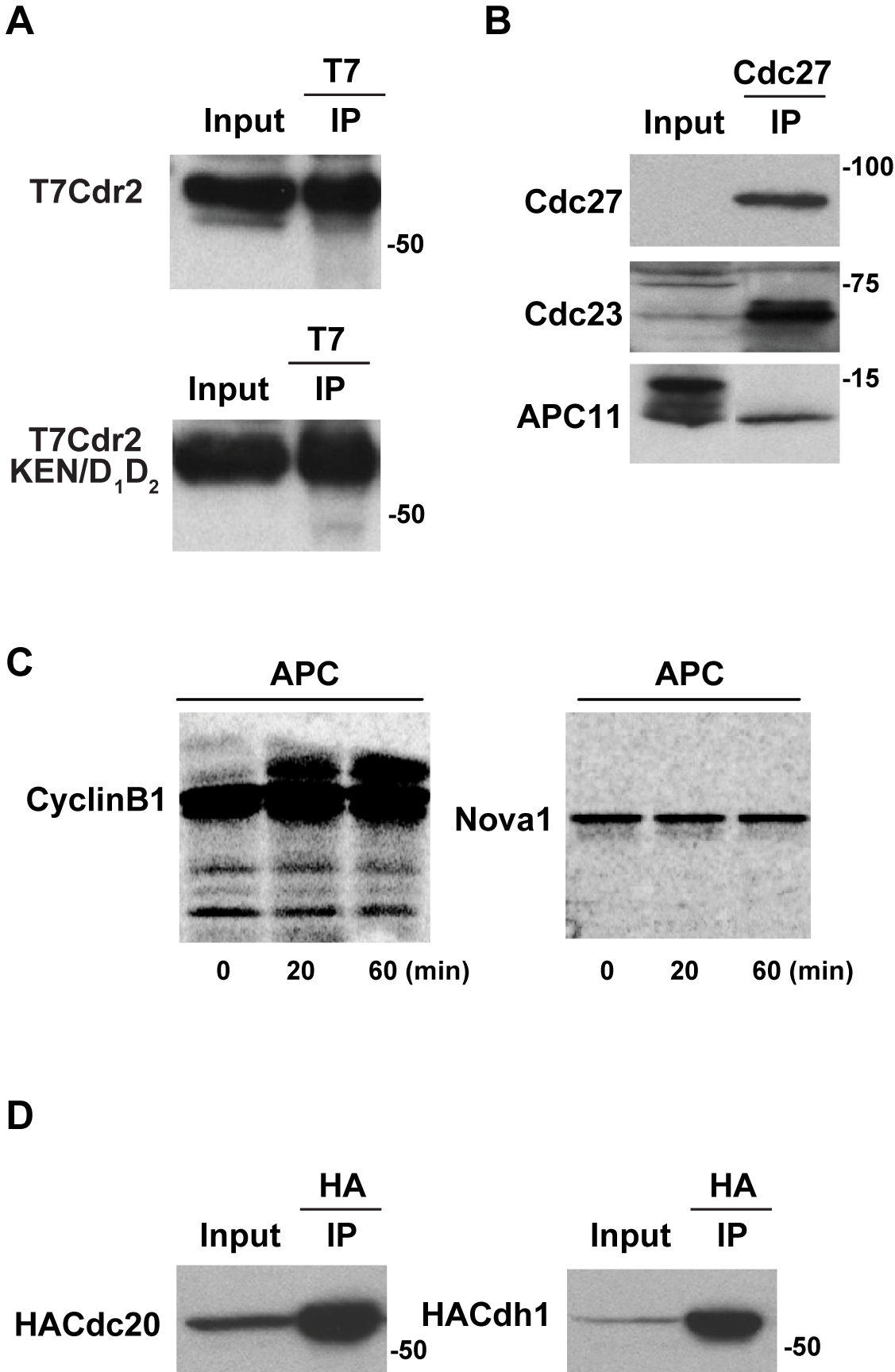

Supplement: Figure S2 — A. Anti-T7 western of input and T7 immunoprecipitates from T7cdr2 (top) and T7cdr2 KEN/D1D2 (bottom) transfected HEK293s. B. Anti-cdc27 immunoprecipitation and western blot with cdc27, cdc23 and APC11 antisera of HEK293s exiting mitosis. C. APC/C in vitro ubiquitination of 35S-cyclinB1 and 35S-Nova1. D. Anti-HA western of HA immunoprecipitation and inputs of HACdc20 and HACdh1 expressing HEK293s. (0.87 MB TIF) [file pone.0010045.s005.tif]

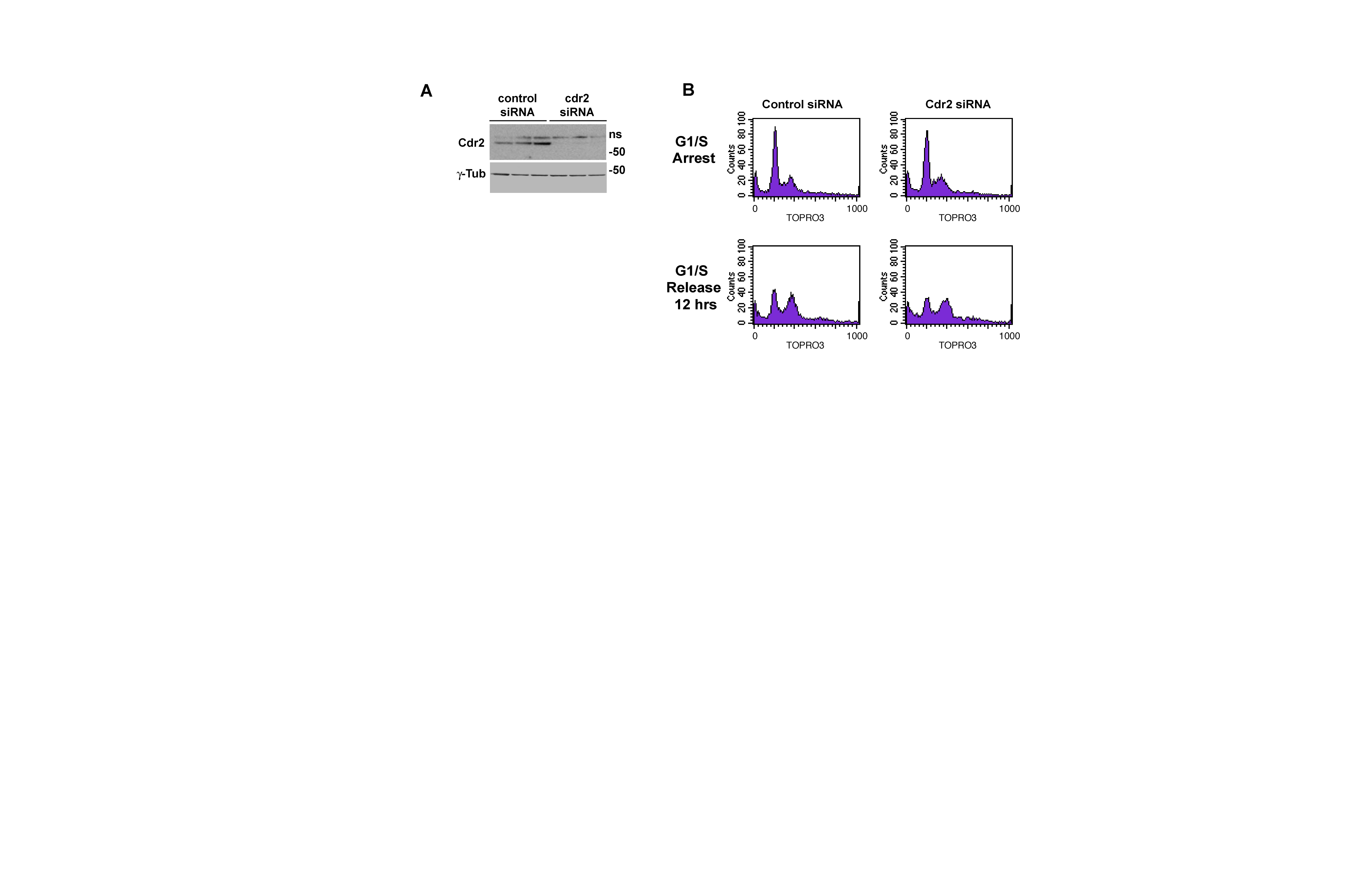

Supplement: Figure S3 — A. Western blot of HeLa extracts transfected with control and cdr2 siRNA pools probed with anti-cdr2 (4F5) and anti-γ-tubulin, showing triplicate experiments. ns indicates non-specific immunoreactive band detected by anti-cdr2 (4F5). B. Flow cytometry of G1S-arrested (top) and G1S-released (12 hour release, bottom) HeLa cells treated with control (left) or cdr2 siRNA pools (right) and stained with TOPRO3 to measure DNA content. (0.60 MB TIF) [file pone.0010045.s006.tif]

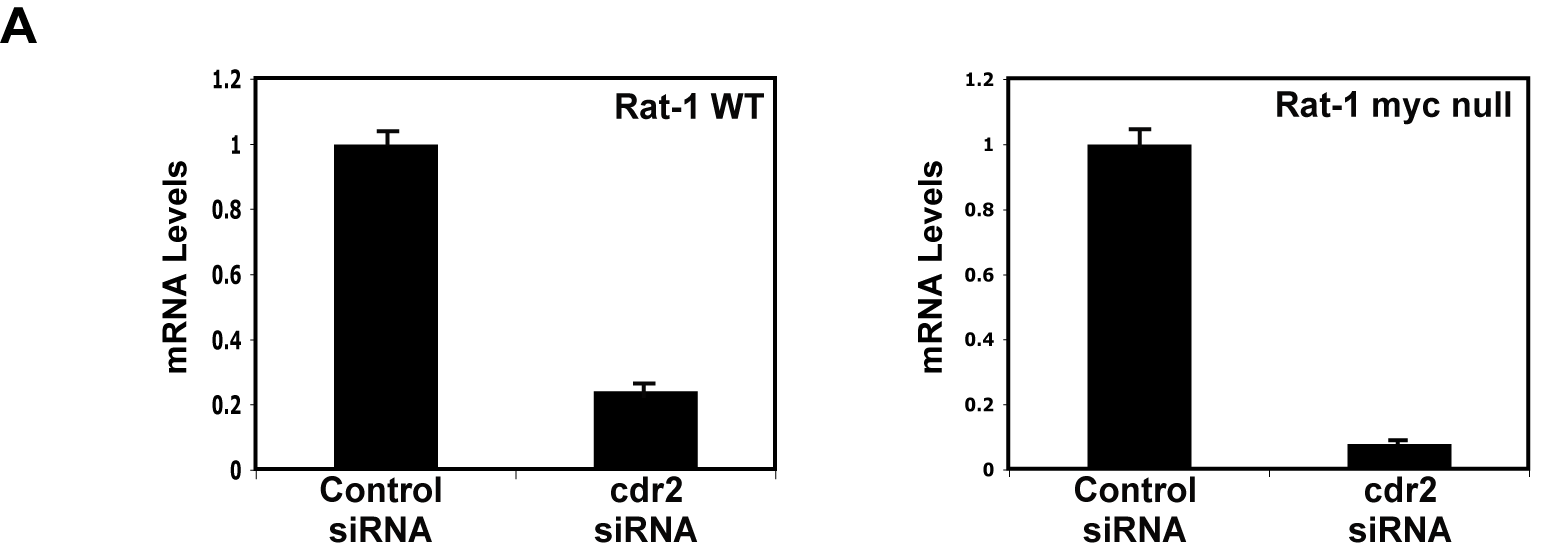

Supplement: Figure S4 — A. qRT-PCR of cdr2 mRNA levels (normalized to GAPDH mRNA) in Rat-1 wild type (left) and Rat-1 c-myc null cells (right) treated with control or rat cdr2 siRNA pools. (0.16 MB TIF) [file pone.0010045.s007.tif]

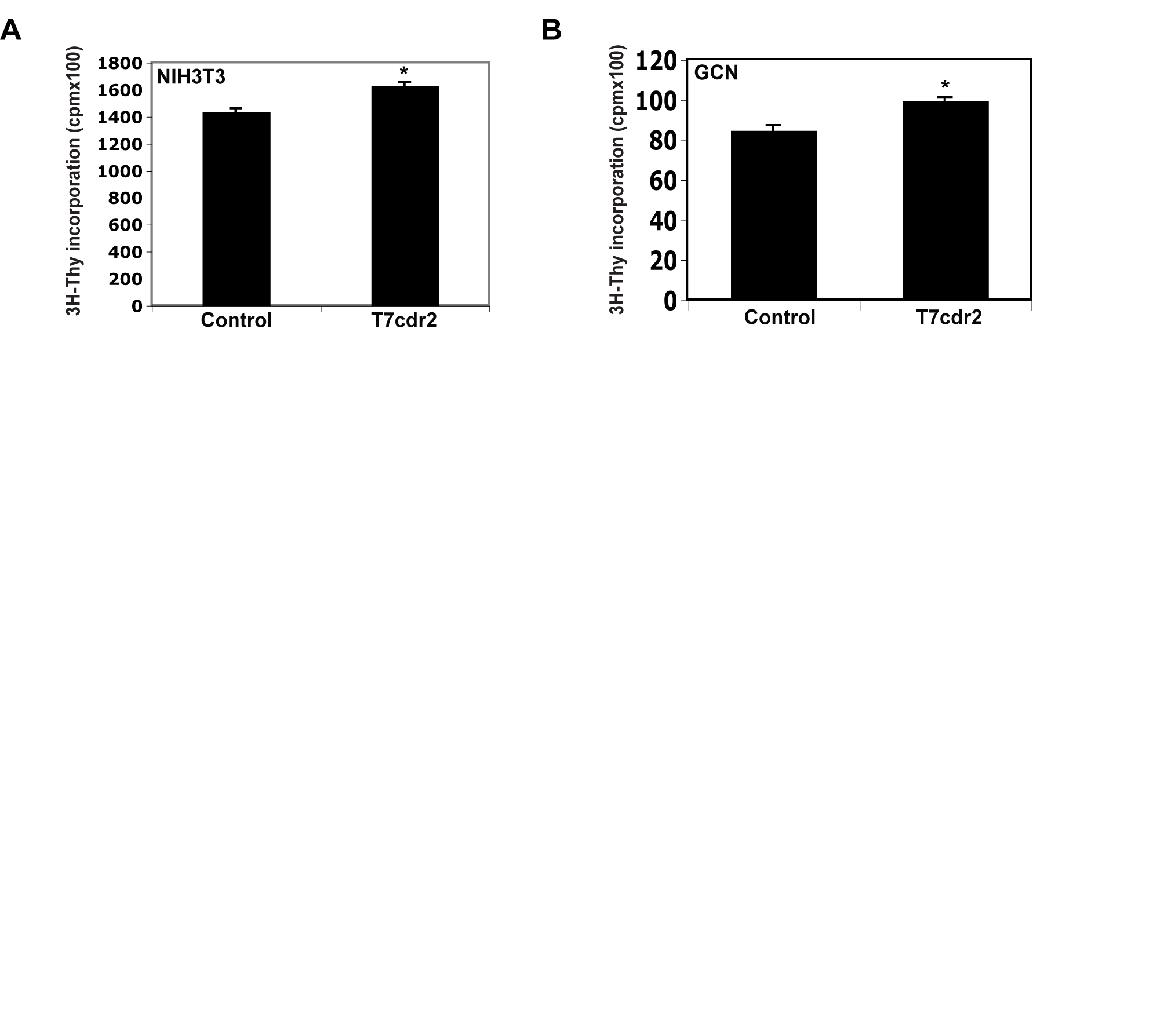

Supplement: Figure S5 — A. NIH3T3 steady state proliferation (3H-thymidine incorporation (cpm)) in stable cell lines expressing control (empty vector) or T7cdr2, *p<0.0001. B. Primary cerebellar granule cell neuron (GCN; 2DIV) steady state proliferation (3H-thymidine incorporation (cpm)) in cells transduced with control or T7cdr2 expressing virus. *p<0.005. (0.36 MB TIF) [file pone.0010045.s008.tif]
